# Variance of the SGK1 Gene Is Associated with Insulin Secretion in Different European Populations: Results from the TUEF, EUGENE2, and METSIM Studies

**DOI:** 10.1371/journal.pone.0003506

**Published:** 2008-11-05

**Authors:** Björn Friedrich, Peter Weyrich, Alena Stančáková, Jianjung Wang, Johanna Kuusisto, Markku Laakso, Giorgio Sesti, Elena Succurro, Ulf Smith, Torben Hansen, Oluf Pedersen, Fausto Machicao, Silke Schäfer, Florian Lang, Teut Risler, Susanne Ullrich, Norbert Stefan, Andreas Fritsche, Hans-Ulrich Häring

**Affiliations:** 1 Department of Internal Medicine, Division of Endocrinology, Diabetology, Vascular Medicine, Nephrology and Clinical Chemistry, University of Tübingen, Tübingen, Germany; 2 Department of Physiology, University of Tübingen, Tübingen, Germany; 3 Department of Medicine, Kuopio University Hospital, Kuopio, Finland; 4 Department of Experimental and Clinical Medicine, Polyclinic Mater Domini, University Magna Graecia of Catanzaro, Catanzaro, Italy; 5 The Lundberg Laboratory for Diabetes Research, Department of Internal Medicine, Sahlgrenska University Hospital, Gothenburg, Sweden; 6 The Steno Diabetes Center, Copenhagen, Denmark; 7 Faculty of Health Science, University of Copenhagen, Copenhagen, Denmark; Mayo Clinic College of Medicine, United States of America

## Abstract

**Hypothesis:**

Serum- and Glucocorticoid-inducible Kinase 1 (SGK1) is involved in the regulation of insulin secretion and may represent a candidate gene for the development of type 2 diabetes mellitus in humans.

**Methods:**

Three independent European populations were analyzed for the association of SGK1 gene (*SGK*) variations and insulin secretion traits. The German TUEF project provided the screening population (N = 725), and four tagging SNPs (rs1763527, rs1743966, rs1057293, rs9402571) were investigated. EUGENE2 (N = 827) served as a replication cohort for the detected associations. Finally, the detected associations were validated in the METSIM study, providing 3798 non-diabetic and 659 diabetic (type 2) individuals.

**Results:**

Carriers of the minor G allele in rs9402571 had significantly higher C-peptide levels in the 2 h OGTT (+10.8%, p = 0.04; dominant model) and higher AUC_C-Peptide_/AUC_Glc_ ratios (+7.5%, p = 0.04) compared to homozygous wild type TT carriers in the screening population. As interaction analysis for BMI×rs9402571 was significant (p = 0.04) for the endpoint insulin secretion, we stratified the TUEF cohort for BMI, using a cut off point of BMI = 25. The effect on insulin secretion only remained significant in lean TUEF participants (BMI≤25). This finding was replicated in lean EUGENE2 rs9402571 minor allele carriers, who had a significantly higher AUC_Ins_/AUC_Glc_ (*TT*: 226±7, *XG*: 246±9; *p* = 0.019). Accordingly, the METSIM trial revealed a lower prevalence of type 2 diabetes (OR: 0.85; 95%CI: 0.71–1.01; p = 0.065, dominant model) in rs9402571 minor allele carriers.

**Conclusions:**

The rs9402571 *SGK* genotype associates with increased insulin secretion in lean non-diabetic TUEF/EUGENE2 participants and with lower diabetes prevalence in METSIM. Our study in three independent European populations supports the conclusion that *SGK* variability affects diabetes risk.

## Introduction

Type 2 diabetes arises when insulin resistance cannot be compensated for with increased insulin secretion owing to a gradual loss of pancreatic beta-cell function [1]. Recently, genome-wide association studies have been undertaken to further investigate the genetic background of type 2 diabetes, revealing that many high risk alleles are located within genes that are linked to beta cell function, including TCF7L2 [2], [3], [4], CDKAL1 [5], [6], [7], [8], SLC30A8 [5], [9], [10], IGF2BP2 [5], HHEX/IDE [6], [9], [11], [12], and CDKN2A/B [13]. Our study therefore focuses on genes that play a role in insulin secretion, using a classical candidate-gene approach.

One interesting candidate for the regulation of insulin secretory function is the serum and glucocorticoid inducible kinase SGK1, which is a ubiquitously expressed serine-threonine kinase in humans that is encoded by the gene *SGK* on chromosome 6q23. SGK1 was originally identified in rodents as a serum and glucocorticoid regulated kinase [14], and was shown to be up-regulated by mineralocorticoids [15], TGF-ß1, and insulin [16]. SGK1 seems to provide an important molecular link between salt and glucose homeostasis, as SGK1^−/−^ knockout mice fed with high-salted chow demonstrated decreased SGK1-dependent cellular glucose uptake [17]. Beyond SGK1 functions in transmembranous glucose transport [18], [19], [20], [21] and insulin signalling [16], SGK1 also plays a role in insulin secretion. In INS-1 cells, *SGK* gene transcription and protein expression is strongly regulated, and SGK1 up-regulates the activity of voltage-gated K^+^ channels, which in turn reduces Ca^++^ influx and inhibits insulin release [22]. Another SGK1-dependent molecular mechanism in insulin secretion is the activation of Na^+^/K^+^-ATPase during plasma membrane repolarisation [23].

Taken together, compelling evidence points to a role of this ubiquitously expressed serine/threonine kinase SGK1 in glucose metabolism, especially in the regulation of insulin secretion. So far, studies on the role of *SGK* genetic variance in human physiology are rather limited. Two studies confirmed an association of *SGK* variability with blood pressure in a German twin population [24] and the cohort of the Scandinavian Malmo Diet and Cancer Study [25]. We conducted our study on *SGK* genetic variance and potential associations with insulin secretory function in the German TUEF cohort and the EUGENE2 consortium (Denmark, Finland, Germany, Italy, and Sweden), as these two European diabetes risk populations were extensively phenotyped for insulin secretion traits at the prediabetic stage. To confirm the relevance of associations found for later onset of type 2 diabetes mellitus, corresponding *SGK* risk alleles were further investigated in the METSIM Trial, which provides a large population-based Finnish cohort for the endpoint diabetes. Analyzing four selected tagging SNPs of *SGK*, the SNP rs9402571 was consistently found to be associated with altered insulin secretion in both prediabetic populations, and was further confirmed to associate with the prevalence of type 2 diabetes mellitus in the population-based cohort.

## Methods

### Participants

Three independent European cohorts were analyzed for *SGK* genetic variance and insulin secretion traits for this study. The TUEF project provided the screening population, while EUGENE2 served as a replication cohort for insulin secretion traits. METSIM is a population-based cohort providing both non-diabetic and type 2 diabetic individuals, and was employed for estimation of *SGK* diabetes-risk alleles. Further details on each of the three study cohorts are provided in the following, with baseline characteristics presented in Table 1.

**Table 1:** Characteristics of the 3 investigated study populations.

	TUEF^a^	EUGENE2^b^	METSIM^c^
	(N = 725)	(N = 827)	(N = 4457)
Gender (m/f)	261/464	344/483	4457/-
Age (years)	37.2±12.5	40.1±10.3	59,5±5.9
BMI	29.3±8.8	26.6±5.0	27,4±4.2
Waist (cm)	93.9±18.4	89.0±13.4	99,1±11.5

Data are presented as means±SD.

a Tuebingen Family Study (South Germany; non-diabetic individuals).

b Finns, Danish, Dutch, Swedish and Germans from the EUGENE2 consortium [27]. Only non-diabetic EUGENE2 participants with complete datasets for AUC_Ins_/AUC_Gluc_ calculations were analyzed.

c METabolic Syndrome In Men (METSIM) cohort from Kuopio (Finland), population-based (non-diabetic and diabetic individuals).

#### TUEF cohort

The TUEF (Tuebingen Familiy Study) cohort includes non-diabetic individuals from southern Germany with increased risks for developing type 2 diabetes (family history of type 2 diabetes, diagnosis of impaired fasting glucose). The study protocol included standard procedures as medical history, physical examination, routine blood tests and oral glucose tolerance test with blood sampling (plasma insulin, plasma glucose, plasma C-peptide) at 0, 30, 60, 90 and 120 min [26]. Informed written consent was obtained from all participants, and all study procedures were approved by the local medical ethic research committee of the Faculty of Medicine at the University of Tuebingen. 1000 TUEF participants were genotyped for *SGK* and phenotyped by OGTT and AUC_CP_/AUC_Glc_ (see below). For further investigation, individuals with one of the following criteria were excluded: taking medications known to affect glucose tolerance, severe diseases (malignancies, cardiovascular or psychiatric disease, etc.), newly diagnosed diabetes, positive GAD antibodies, and one or more missing parameters needed for AUC_CP_/AUC_Glc_ calculation. This method of elimination resulted in a final study cohort of 725 non-diabetic individuals.

#### EUGENE2 consortium

Five different European clinical diabetes centres contributed non-diabetic offspring of patients with type 2 diabetes to the EUGENE2 (European network on Functional Genomics of Type 2 Diabetes) consortium, including the Lundberg Laboratory for Diabetes Research (Göteborg, Sweden), the Polyclinic Mater Domini of the University Magna Graecia (Catanzaro, Italy), the Steno Diabetes Center (Copenhagen, Denmark), the Kuopio University Hospital (Kuopio, Finland), and the Tübingen University Hospital (Tübingen, Germany). All study participants underwent a standard medical history, routine laboratory testing, assessment of social issues (alcohol consumption, activity, smoking status), and an OGTT. Informed written consent was obtained of all participants, and the local ethics committees approved the study protocol at the different centres. Further details about the EUGENE2 consortium are provided elsewhere [27].

#### METSIM cohort

The Finnish cohort comprised 4457 male participants, aged from 50 to 70 years. Among them, 43.4% had a family history of diabetes. The primary aim of the ongoing METSIM (METabolic Syndrome In Men) trial is to investigate the effects of SNPs in genes of interest on the risk of type 2 diabetes and cardiovascular disease in a random sample of Finnish men, living in the town of Kuopio (population 95,000), in eastern Finland [4]. The WHO criteria in 1999 for diabetes mellitus were used for classification of the METSIM participants, based on fasting plasma glucose and 2-hour post-load glucose levels in an OGTT [28] conducted at baseline. Among the 4457 participants, 659 had known or newly diagnosed diabetes and 3798 were non-diabetic. The protocol includes a 1-day visit to the Clinical Research Unit of the University of Kuopio. This study was approved by the Ethics Committee of the University of Kuopio and was in accordance with the Helsinki Declaration.

### Body composition and body fat distribution

Body mass index (BMI) was calculated as weight divided by the square of height (kg/m^2^). Waist and hip circumferences were measured in the upright position.

### Analytical procedures

Blood glucose was determined using a bedside glucose analyzer (Yellow Springs Instruments, Yellow Springs, CO, USA). Plasma insulin was determined by microparticle enzyme immunoassay (Abbott Laboratories, Tokyo, Japan) for both the TUEF and EUGENE2 cohorts. Plasma C-peptide was determined by radioimmunoassay (Byk-Sangtec, Dietzen bach, Germany).

### Oral glucose tolerance test (OGTT)

The OGTT was performed according to the recommendations of the World Health Organization after a 12 h fasting period [28]. Blood glucose, insulin (all centres), and C-peptide (TUEF cohort only) plasma levels were determined at 0, 30, 60, 90, and 120 min.

### Insulin sensitivity and insulin secretion

Insulin sensitivity was calculated from glucose and insulin values obtained during the OGTT, as proposed by Matsuda and DeFronzo [29]. Estimation of insulin secretion by the OGTT parameters was obtained by C-peptide levels measured at 30 min during the OGTT. Alternatively, insulin secretory function was calculated by the ratio of the area under the curve (AUC) of C-peptide (AUC_CP_; TUEF cohort) or insulin (AUC_Ins_; EUGENE2 cohort) plasma levels, referred to as the AUC of plasma glucose (AUC_Glc_), using the trapezoidal approximation rule for AUC calculation [30].

### SNP Genotyping

The *SGK* (NM_005627) gene on chromosome 6q23 was subjected to Hap Map analysis in the CEU population (Utah residents with ancestry from Northern and Western Europe; data release 21a/Jan07). Tagging SNPs were selected using the TagSNP Picker software (settings: multimarker mode MAF>0.05, r^2^>0.9), screening the complete *SGK* gene, 10 kb of the promoter, and 1.5 kb downstream of the 3′ untranslated region. Genotyping of tagging SNPs was accomplished by means of the TaqMan® assay and an ABI Prism 7500 sequence detection system (Applied Biosystems, Foster City, CA, USA). Genotyping quality was tested by including three known controls of each genotype in each assay.

### Data Analysis

Unless otherwise noted, data are stated as means±SEM. Comparison between genotype groups was performed using logarithmically transformed data for non-normally distributed parameters in ANOVA and *t*-tests. Each effect was adjusted for relevant covariates using mixed linear models. Parameters that remained non-normally distributed despite log transformation were directly analyzed by a non-parametric (Wilcoxon) test. Association with diabetes (METSIM) was tested using logistic regression analysis. Hardy-Weinberg equilibrium was tested with the χ^2^ test. A *p*-value≤0.05 was considered to be statistically significant. The software package JMP (SAS Institute Inc, Cary, NC, USA) and SPSS 14.0 for Windows (SPSS Inc., Chicago, IL, USA) were used for statistical data analysis. The software JLIN (Western Australian Institute for Medical Research) was used for calculation of linkage disequilibrium [31].

## Results

### Genetic analysis of the human *SGK* gene

**Figure 1:**
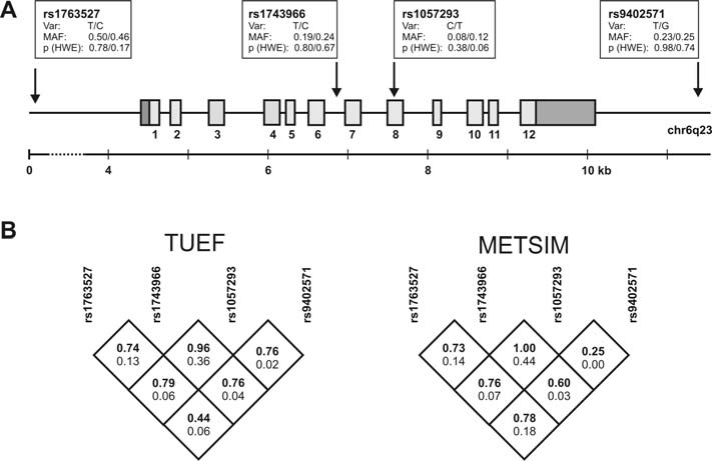
The gene encoding human serum- and glucocorticoid-regulated kinase 1 (SGK) on chromosome 6q23. A) Exons (light grey) and untranslated regions (dark grey) are indicated. Four tagging SNPs were selected by HapMap analysis considering the SGK gene ±10 kb upstream of the first translational start codon and 1.5 kb downstream of the 3′ untranslated region. SNP information is provided in boxes (var = variation; MAF = minor allele frequency; HWE = Hardy-Weinberg equilibrium, χ^2^ test) for populations investigated for all tagging SNPs (South German TUEF cohort/Finnish METSIM cohort). B) Linkage disequilibrium analysis of selected tagging SNPs for the TUEF and METSIM population: D' (in bold, upper numeral) and r^2^ (bottom numeral).

The human *SGK* gene comprises 12 exons spanning 5.6 kb on chr6q23. Analysis of a 17 kb region of the *SGK* locus by Tag SNP Picker revealed five tagSNPs in the HapMap, each with a minor allele frequency (MAF) of >0.05 in the CEU population. Four SNPs (rs1763527, rs1743966, rs1057293, and rs9402571) were finally selected for genotyping, covering 83% of all SNPs fulfilling the inclusion criteria mentioned above. Both rs1743966 (intron 6) and rs1057293 (exon 8; synonymous: Asp/Asp) are located within the *SGK* gene, while rs1763527 (promoter) and rs9402571 (1429 bp downstream of the *SGK* 3′UTR) tag the regions surrounding *SGK*. MAF and Hardy-Weinberg-Equilibrium test results of all tagging SNPs are indicated for the TUEF and EUGENE2 cohort in Fig. 1A, with linkage disequilibrium (LD) analysis provided in Fig. 1B.

### *SGK* association analyses in the TUEF screening cohort

There was no association between genotypes, anthropometrics, and insulin sensitivity in the south German screening cohort, which was investigated for all four tagging SNPs (data not shown). Most consistently, two different indices of insulin secretion differed significantly among rs9402571 genotypes (Table 2). Minor allele carriers of rs9402571 had significantly higher C-Peptide levels in the 2 h OGTT than individuals with the wild type allele (+10.8%, p = 0.04, dominant model), after adjustment for age, gender, BMI, and insulin sensitivity. This genotype also associated with better AUC_C-Peptide_/AUC_Glc_ ratios (+7.5%, p = 0.04; dominant model, adjusted for age, BMI and gender; Table 2).

**Table 2:** Metabolic Traits of the German Cohort according to rs9402571 genotype.

SNP Genotype	______rs9402571______			*p_1_*	*p_2_*
	TT	TG	GG		
N	434	251	40	-	-
Age (y)	37.0±0.6	37.1±0.8	39.6±2.2	0.44	0.62
BMI (kg/m^2^)	29.0±0.4	30.0±0.6	28.9±1.3	0.24^a^	0.20
Fasting glucose (mM)	5.10±0.03	5.05±0.04	5.08±0.09	0.10^b^	**0.0376**
Glucose, 120 min OGTT (mM)	6.19±0.08	6.11±0.11	6.33±0.29	0.31^b^	0.16
Insulin sensitivity, OGTT (U)	17.1±0.5	16.9±0.8	14.1±1.4	0.12^b^	0.64
C-Peptide, 30 min OGTT (pM)	2020±43	2153±60	2239±158	0.12^c^	**0.0398**
AUC_C-peptide_/AUC_glucose_, OGTT (·10^−9^)	318±5	335±7	342±18	0.09^b^	**0.0416**

Data are given as means±SEM; p_1_–additive model; p_2_–dominant model. AUC–area under the curve; OGTT–oral glucose tolerance test; SNP–single nucleotide polymorphism.

a adjusted for age and gender.

b adjusted for age, gender and BMI.

c adjusted for age, gender, BMI and insulin sensitivity.

### Interaction analysis and replication in EUGENE2

**Figure 2:**
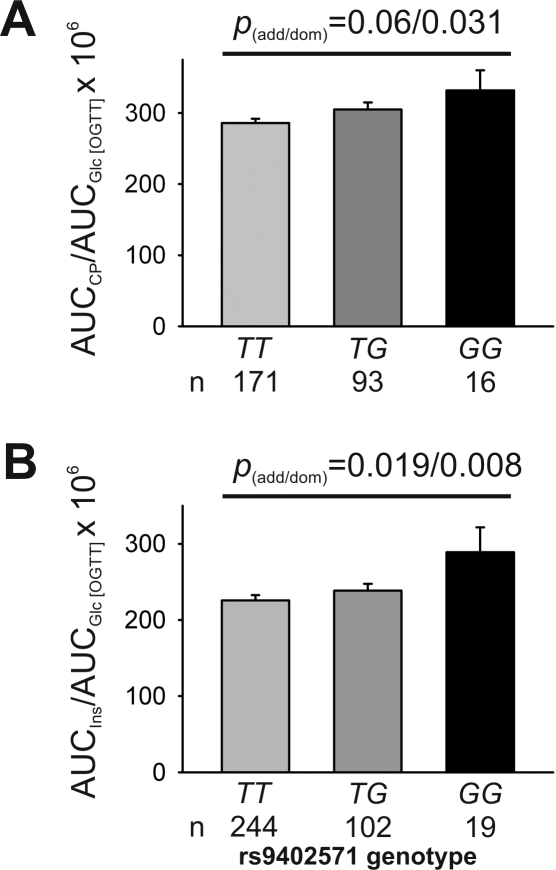
Insulin secretion of the TUEF and EuGene2 Cohort, according to rs9402571 genotype in lean subjects (BMI<25). A) TUEF cohort: The ratio of the area under the curve (AUC) of C-Peptide values (AUC_CP_) to AUC glucose (AUC_Glc_) in the OGTT was adjusted for age, gender and insulin sensitivity (Matsuda). B) EUGENE2 cohort: the ratio of AUC insulin (AUC_Ins_) to AUC glucose (AUC_Glc_) was adjusted for centre, familial relationship, age, gender and insulin sensitivity. Only individuals with a BMI<25 kg/m^2^ were included for both analysis. Data are presented as means±SEM. p-values are shown for both the additive and dominant model.

An interaction analysis for the trait of insulin secretion was performed. BMI×rs9402571 genotype was revealed to be significantly relevant for the endpoint insulin secretion in TUEF, independent of which calculation model for insulin secretion was used (p = 0.04, both models) and of whether BMI was treated as a continuous or discrete variable with BMI cut-off points ranging from 24 to 28 kg/m^2^. We therefore stratified the cohort for BMI, using a cut off point of BMI = 25 to distinguish lean from overweight or obese individuals, and did a reanalysis for insulin secretion for both subgroups. The effect on AUC_CP_/AUC_Glc_ was only significant in the lean TUEF subgroup (BMI≤25: *TT*: 318±5, *TG*: 335±7, *GG*: 342±17; *p* = 0.031; dominant model, adjusted for age, gender, and insulin sensitivity; Fig. 2A), while there was no association with insulin secretory function in overweight to obese study participants (BMI>25; p>0.24, all; additive and dominant models; data not shown). To replicate our findings of the association between rs9402571 and insulin secretion, the EUGENE2 cohort was genotyped for rs9402571 and the AUC_Ins_/AUC_Glc_ ratio was calculated for each genotype. Again, AUC_Ins_/AUC_Glc_ was significantly higher (*TT*: 226±7, *TG*: 239±9, *GG*: 289±33; *p*  = 0.019 for the dominant model; adjusted for study centre, familial relationship, age, gender, and insulin sensitivity; Fig. 2B) in lean (BMI≤25) minor allele carriers of rs9402571, while there was no significant association in overweight to obese individuals (BMI>25: p>0.44; data not shown).

### Diabetes risk analysis in the METSIM population-based cohort

**Figure 3:**
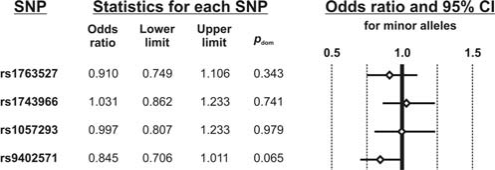
Diabetes risk (METSIM study) according to SGK genetic variability. The odds ratio, 95% confidence intervals (CI) and forest plot for the endpoint “type 2 diabetes” are presented for the minor alleles of each SGK tagging SNP. N = 658 diabetic and N = 3746 non-diabetic subjects of the Finish METSIM study cohort were investigated. The p-values were calculated for a dominant model. SNP = single nucleotide polymorphism.

The METSIM cohort was genotyped for all four selected tagging SNPs to analyze *SGK* genetic variance for the endpoint diabetes (type 2) in a population-based cohort. The minor allele of rs9402571, which was found to associate with a higher insulin secretion in the screening (TUEF) as well as the replication (EUGENE2) populations, associated almost significantly (p = 0.065; adjusted for age, family history of diabetes, and BMI) with a lower prevalence of type 2 diabetes (odds ratio: 0.845; 95%CI: 0.706–1.011), comparing the 659 diabetic patients with the non-diabetic 3798 METSIM participants. In contrast, there was no significantly altered odds ratio in regard to the three other genotyped *SGK* tagging SNPs rs1763527, rs1743966, and rs1057293 (Fig. 3).

## Discussion

This is the first study to investigate the role of *SGK* (Serum- and Glucocorticoid-inducible Kinase 1) genetic variance in two independent prediabetic diabetes risk populations (TUEF and EUGENE2), including a confirmatory analysis in a third independent, older population that includes both diabetic and non-diabetic individuals (METSIM). *SGK* is an attractive candidate gene for type 2 diabetes mellitus onset in humans, as SGK1 exerts pleiotropic effects on glucose metabolism and insulin secretion in various cellular and animal models [16], [22], [23].

In the screening population (TUEF; N = 725), we found a significant association of rs9402571 with glucose-induced insulin secretion in various estimation models based on oral glucose tolerance testing. This association was reproducible in a subgroup that was additionally phenotyped by an intravenous glucose tolerance test (data not shown). After confirming a significant interaction between BMI and rs9402571 for the endpoint insulin secretion, BMI stratification (BMI cut off: 25) was introduced in our analysis, revealing that only lean individuals were affected by the rs9402571 minor allele. This genotype-phenotype association was also restricted to lean individuals in the replication cohort from the EUGENE2 consortium (N = 827), comprising individuals from Sweden, Italy, Denmark, Finland, and Germany. Interestingly, the rs9402571 allele that associates with higher insulin secretion in TUEF and EUGENE2 accordingly associates with lower diabetes prevalence in the METSIM trial. Despite the fact that SGK1 has a regulatory function for glucose transporter expression and translocation [18], [20], [21], no association of the four investigated *SGK* tagging SNPs with insulin sensitivity was found in the TUEF screening population or in the EUGENE2 study. One could suggest that the *SGK* rs9402571 SNP was not detected in the genome-wide association (GWA) studies due to its dependence on BMI, as the GWA analyses did not stratify for BMI. Interestingly, a genome-wide linkage analysis identified the gene locus 6q23, which also includes *SGK*, to be associated with BMI progression in participants of the Framingham Heart Study [32].

The increase in insulin secretion reported here (10.8% in TUEF; 8.8% in EUGENE2) within lean rs9402571 G allele carriers was remarkable compared to wild type genotype, and may indicate a protective genotype against type 2 diabetes. This protective rs9402571 *SGK* genotype may become blurred in overweight or obese individuals, as environmental factors may dominate compared to the *SGK* genotype. However, our analysis in the METSIM trial assumed that the protective rs9402571 *SGK* genotype effect obviously persists even in a population-based cohort containing individuals 20 years older than those from the TUEF and EUGENE2 studies. Therefore, although the number of study participants was limited after BMI stratification in both TUEF and EUGENE2, our findings in three independent European populations allow the assumption that the *SGK* rs9402571 genotype is protective against type 2 diabetes. Further studies on the role of SGK1 in human insulin secretion and diabetes onset are therefore needed.
